# HIV携带者并发肺腺癌1例及文献复习

**DOI:** 10.3779/j.issn.1009-3419.2013.02.10

**Published:** 2013-02-20

**Authors:** 晶 赵, 达 姜, 荣凤 刘, 欣 李

**Affiliations:** 050000 石家庄，河北医科大学第四医院肿瘤内科 Department of Oncology, the Fourth Hospital of Hebei Medical University, Shijiazhuang 050000, China

## 1 临床资料

患者，男，57岁，河北省衡水市景县人，于2012年10月11日收入河北医科大学第四医院肿瘤内科诊治。入院前2月患者因受凉出现咳嗽，咳白色稀痰，伴胸闷、胸痛、气短、乏力，平卧位时症状加重。无痰中带血及咯血，无发热盗汗，无声嘶。口服感冒药及静脉输注消炎药（具体不详）后无明显缓解，就诊于景县人民医院，胸部CT检查提示：右肺中叶团块状软组织密度影，边缘欠清；右肺炎症；右侧胸腔积液（图 1）。未行正规治疗，1月前就诊于河北医科大学第四医院胸外科，胸水脱落细胞学检查：找到癌细胞，考虑肺腺癌。行顺铂+吉西他滨联合化疗1周期，同时博来霉素60 mg胸腔注药，末次化疗时间为2012年9月9日。后就诊于河北医科大学第四医院肿瘤内科，患者拒绝行表皮生长因子受体（epidermal growth factor receptor, EGFR）等分子靶标检测及酪氨酸激酶抑制剂（tyrosine kinase inhibitor, TKI）靶向治疗。根据美国国立综合癌症网络（National Comprehensive Cancer Network, NCCN）指南结合患者情况，考虑培美曲塞更具优势且副作用较小，给予培美曲塞+顺铂方案化疗1周期，末次化疗时间为2012年10月14日。化疗后患者出现Ⅰ度骨髓抑制，消化道反应轻微，化疗后患者未定期返院。2012年12月20日患者出现头痛、头晕，伴呕吐，景县人民医院查头颅CT：颅内多发不规则低密度影，考虑转移，再次就诊于河北医科大学第四医院肿瘤内科，给予脱水降颅压治疗，患者同意口服吉非替尼片（印度版）靶向治疗（250 mg，1次/日）。治疗后上述症状缓解，无皮疹等副反应，目前仍在随访中。

**1:**
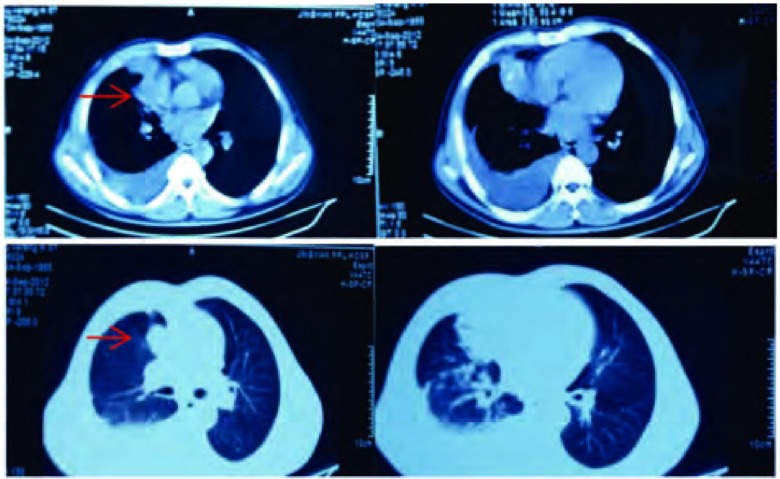
景县人民医院胸部CT（2012年9月4日）：右肺中叶肿块并右侧胸腔积液 Chest CT of King County People's Hospital (2012-09-04): right middle lobe mass and right pleural effusion

既往自诉于2008年因拔牙感染人类免疫缺陷病毒（human immunodeficiency virus, HIV），并于衡水市疾控中心确诊为HIV阳性，此后规律口服拉米夫定（100 mg，1次/日），齐多夫定（300 mg，2次/日）和奈韦拉平（200 mg，2次/日）。每3个月到疾控中心领取药品及化验相关指标。依据艾滋病诊疗指南，接受高效联合抗逆转录病毒治疗（highly active antiretroviral therapy, HAART）的患者治疗1年以上且病情稳定，每年行2次CD4^+^T淋巴细胞数检测。衡水市疾控中心每年为此患者免费行2次CD4^+^T淋巴细胞数目及1次HIV病毒载量测定。2012年4月患者CD4^+^T淋巴细胞数为559个/mm^3^，HIV病毒载量为10拷贝/mL；2012年7月CD4^+^T淋巴细胞数为402个/mm^3^。但患者这两次CD4^+^T淋巴细胞数目及1次HIV病毒载量测定均为发现肺癌之前数据。化疗2个疗程后在河北医科大学第四医院检查（2012年11月13日）T淋巴细胞增殖活性，检测报告：CD3阳性细胞率38%，CD4阳性细胞率16%，CD8阳性细胞率18%，NK阳性细胞率24%，CD4/CD8为0.89，CD19阳性细胞率8%；检测结果：T细胞数量均减少，NK细胞数量减少，T4/T8比值倒置。发生脑转移后检查（2012年12月31日）T淋巴细胞增殖活性，检测报告：CD3阳性细胞率50%，CD4阳性细胞率20%，CD8阳性细胞率32%，NK阳性细胞率29%，CD4/CD8为0.63；检测结果：T细胞总量和T4细胞数量减少，T8细胞数量增高，NK细胞数量减少，T4/T8比值倒置。CD19阳性细胞率8%，B细胞数量正常。

患者吸烟40年，每日约20支，现仍未戒烟。偶少量饮酒，否认性病、冶游史，否认吸毒及输血史。无肝炎、梅毒等传染病史。28岁结婚，育2子1女，子女及配偶健康状况良好，检查均未感染HIV病毒。

## 2 讨论

HIV感染者/获得性免疫缺陷综合症（Acquired Immune Deficiency Syndrome, AIDS）患者易患各种恶性肿瘤，其中卡波西肉瘤、恶性淋巴瘤、子宫颈癌在亚洲以外地区的HIV/AIDS人群中发生率高，称为艾滋病相关肿瘤。近年来，由于艾滋病发病率的增加，非HIV相关肿瘤（肺癌、结肠癌、乳腺癌、胃癌、肝癌多见）也日益受到关注。研究^[1]^报道1991年-1995年和2001年-2005年，艾滋病相关肿瘤降低了3倍（34, 587 *vs* 10, 325; *P*＜0.001），而非艾滋病相关肿瘤增加了约3倍（3, 193 *vs* 10, 059; *P*＜0.001）。

1984年，Irwin等^[2]^首先在艾滋感染者中发现1例转移性非小细胞肺癌患者。2004年-2007年美国34个洲HIV携带者中，共发生2, 191例非艾滋病相关肿瘤，其中肺癌454例^[1]^。检索发现，1980年-2007年HIV阳性合并肺癌患者共3, 963例。对Brescia研究^[3]^、GICAT研究^[4]^等进行分析，显示HIV合并肺癌发病中位年龄为46.9岁，男性居多，男女比例为5-10:1^[5]^。还有证据^[6]^表明，HIV阳性患者患肺癌的年龄比未感染HIV患者提前10年以上，其中位CD4^+^T淋巴细胞数常多于200个/μL。HIV阳性合并肺癌患者中非小细胞肺癌占86%-94%，以腺癌居多，约占30%-52%。而且，大约70%-90%的患者被诊断为肺癌即处于局部晚期或进展期^[7]^，患者中位生存时间5个月。

重度吸烟被认为是HIV阳性患者发生肺癌的主要危险因素之一^[6]^，即使在调整吸烟密度及强度等因素的情况下，肺癌的发生率仍是普通人群的2倍-4倍，HIV可增加吸烟的致癌作用。其它诸如HIV持续性的致癌作用；反复肺部感染；HIV引起的免疫缺陷及HIV相关免疫监视作用降低等也使肺癌的发生机率大增。新的抗逆转录治疗药物，雷特格韦相对安慰剂组有更高的肿瘤发生风险^[8]^，提示抗HIV药物有一定的致癌作用。

实验研究^[9]^表明，*HIV tat*基因产物可增加一些原癌基因如*c*-*myc*、*c*-*fos*、*c*-*jun*的表达，在肺腺癌患者中还能下调肿瘤抑制基因p53的表达。此外，在HIV感染合并肺癌患者中，微卫星改变相比未感染HIV的肺癌患者增加了6倍^[10]^，HIV导致的基因不稳定增加了肺癌的发生。肺感染引起的慢性炎症也可能加强吸烟患者患肺癌机率，然而，CD4^+^T淋巴胞数减少及HIV病毒载量增加与肺癌的发生无明显关联^[11]^。

此病例在河北医科大学第四医院肿瘤内科为首次发现，具有一定临床意义。鉴于我国HIV现处于高发阶段，且HIV/AIDS易并发恶性肿瘤的机会增大，因此，临床上会有越来越多的此类病例，对于HIV合并肺癌患者的治疗来说，目前尚缺少相关指南。笔者认为，在患者免疫功能状态正常及一般情况较好的情况下，应遵循非小细胞肺癌治疗的一般原则。对于早期患者，根治性手术仍作为首选。而对无局部治疗机会的患者，则选择放化疗及分子靶向药物治疗等综合治疗。尽管*EGFR*突变对肺腺癌的发生有密切关系，但Chinn等^[12]^研究显示AIDS与*EGFR*突变联系不明显。此外，在治疗期间应注重监测免疫功能，及时调整治疗策略。

有证据表明，在未行HAART或HAART早期阶段，HIV阳性肺癌患者的生存时间明显短于未感染HIV患者。最近的研究^[13]^显示，在HAART期间，两组生存率无明显差异。对于合并HIV阳性的非小细胞肺癌经积极的治疗后可以获得与HIV阴性患者类似的生存期。导致这个结果可能是因为HAART治疗减少了机会性感染的机会，提高了生存状态，有助于患者接受更多的化疗。某些抗HIV药物有抑制肿瘤的作用，蛋白酶抑制剂奈非那韦能够提高对卡波西肉瘤细胞的免疫监视，通过增加内质网压力促进肿瘤细胞死亡^[14]^，间接或直接抑制肿瘤转移。肿瘤、宿主、HIV病毒、抗HIV药物与抗肿瘤药物之间可能存在复杂的相互关系（图 2），抗HIV治疗除了抗HIV的作用，还可抑制肿瘤转移，与抗肿瘤治疗有协同作用，同时，对改善宿主免疫功能有一定程度影响。而抗肿瘤治疗除抑制肿瘤生长外，可引起免疫细胞减少，削弱患者免疫功能，利于HIV病毒复制。鉴于抗HIV药物奈非那韦在体外选择性抑制人类表皮生长因子受体2（human epidermal growth factor receptor-2, HER2）阳性乳腺癌细胞生长的事实^[15]^，有理由相信，HAART治疗同样也可能使HIV阳性肺癌患者获益。本例患者也正是在积极抗HIV治疗的同时实施综合治疗，生活质量明显提高。

**2:**
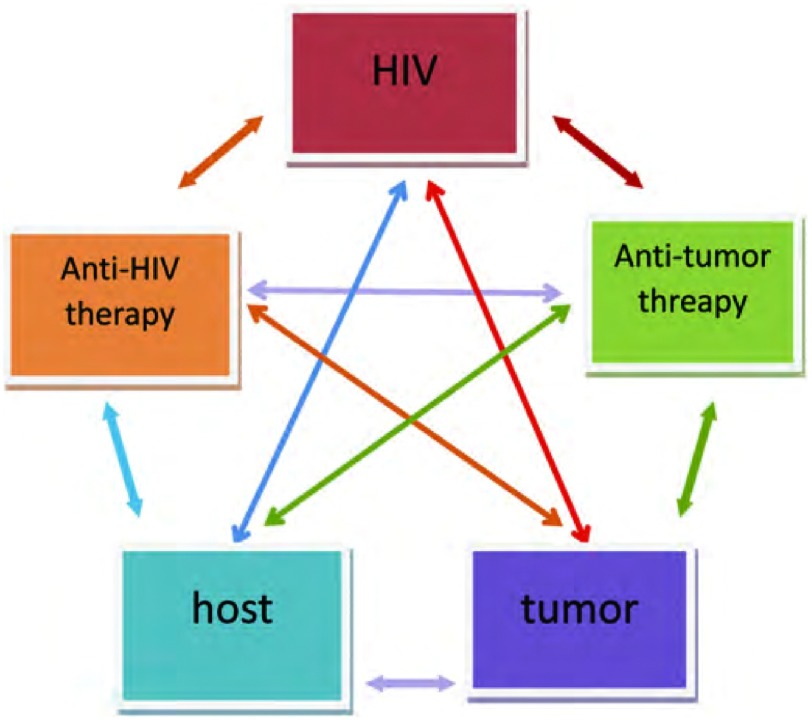
肿瘤、宿主、HIV病毒、抗HIV治疗与抗肿瘤治疗之间可能存在的复杂关系 Tumor, host, HIV virus, anti-HIV therapy and antitumor therapy that may exist among the complex interrelationships

抗HIV药物与化疗药物的相互作用可影响疗效^[16]^。研究证实，细胞色素P450（Cytochrome P450, CYP450）可降解依托泊苷、紫杉醇、长春新碱及盐酸厄洛替尼、吉非替尼等化疗药物，蛋白酶抑制剂利托那韦可通过抑制CYP450进而影响化疗药物代谢，增加化疗药物作用。当然，药物间的相互作用是极其复杂的。有文献报道利托那韦还能将诸如紫杉醇、长春新碱等化疗药物泵出细胞外，具有减弱化疗药物的作用。除此之外，临床工作中也应关注抗HIV药物自身毒副作用对患者的影响。核苷类似物如齐多夫定可加重化疗患者的骨髓抑制；齐多夫定联合拉米夫定是脱氧核糖核酸（deoxyribonucleic acid, DNA）修复链的终止者，具有基因毒性。因此，对抗HIV联合化疗患者，应尽可能避免联合治疗带来的毒性相加作用，进而使患者生活质量下降、生存期缩短。

需要指出的是，临床并未推荐正在进行化疗的HIV阳性患者常规预防机会性感染。本例患者化疗后出现Ⅰ度骨髓抑制及轻微消化道反应，尚可耐受，临床上并未对可能发生的感染进行预防，考虑到患者免疫缺陷，化疗后患者T淋巴细胞数目减少，T4/T8比值倒置，易发生各种机会向感染。因此，化疗过程中给予胸腺肽等免疫增强剂治疗，化疗后给予重组人粒细胞刺激因子治疗，同时嘱其预防感冒，定期监测血常规等相关指标。笔者认为机会性感染及各种可能的感染始终是HIV阳性合并肺癌患者的危险因素，甚至是致命。因此治疗期间，应常规监测CD4^+^、CD8^+^T淋巴细胞计数。
